# 9-cis-Retinoic Acid Improves Disease Modelling in iPSC-Derived Liver Organoids

**DOI:** 10.3390/cells14130983

**Published:** 2025-06-26

**Authors:** Mina Kazemzadeh Dastjerd, Vincent Merens, Ayla Smout, Rebeca De Wolf, Christophe Chesné, Catherine Verfaillie, Stefaan Verhulst, Leo A. van Grunsven

**Affiliations:** 1 Liver Cell Biology Research Group, Vrije Universiteit Brussel, 1090 Brussel, Belgium; mina.kazemzadeh.dastjerd@vub.be (M.K.D.);; 2 Biopredic International SARL, Parc de la Bretech, 35760 Saint Grégoire, France; c.chesne@wepredic.com; 3 Stamcelinstituut Leuven, KU Leuven, 3000 Leuven, Belgium

**Keywords:** liver fibrosis, DILI, hepatic stellate cell activation, hepatocytes, drug metabolism, iPSC

## Abstract

Liver fibrosis majorly impacts global health, necessitating the development of in vitro models to study disease mechanisms and develop drug therapies. Relevant models should at least include hepatocytes and hepatic stellate cells (HSCs) and ideally use three-dimensional cultures to mimic in vivo conditions. Induced pluripotent stem cells (iPSCs) allow for patient-specific liver modelling, but current models based on iPSC-derived hepatocytes (iHepatocytes) and HSCs (iHSCs) still lack key functions. We developed organoids of iHepatocytes and iHSCs and compared them to HepaRG and primary HSC organoids. RNA sequencing analysis comparison of these cultures identified a potential role for the transcription factor RXRA in hepatocyte differentiation and HSC quiescence. Treating cells with the RXRA ligand 9-cis-retinoic acid (9CRA) promoted iHepatocyte metabolism and iHSC quiescence. In organoids, 9CRA enhanced fibrotic response to TGF-β and acetaminophen, highlighting its potential for refining iPSC-based liver fibrosis models to more faithfully replicate human drug-induced liver injury and fibrotic conditions.

## 1. Introduction

Liver fibrosis is a major health burden affecting a considerable portion of the global population [1]. In a healthy liver, hepatocytes line the hepatic sinusoids and contribute to lipid, carbohydrate, and bile acid metabolism while also being responsible for the elimination of toxic compounds. Hepatic stellate cells (HSCs), located between the hepatocytes and the sinusoids, are responsible for the storage of vitamin A in the form of retinyl esters. Upon liver damage—whether due to lipid overload, viral infection, or drug toxicity—a multitude of signals from the liver will induce the differentiation of HSCs from their vitamin A storing quiescent state to myofibroblasts [2]. These myofibroblasts will produce an extracellular matrix as scar tissue around the site of liver injury. In the context of chronic liver injury, excessive extracellular matrix deposition leads to pathological liver fibrosis, which disrupts liver architecture and function. If left untreated, fibrosis can progress to advanced liver diseases, including cirrhosis and hepatocellular carcinoma.

The development of accurate in vitro liver fibrosis models is crucial for advancing our understanding of the disease and for the development of novel anti-fibrotic therapies. These models must at least include both hepatocytes and HSCs and ideally be cultured in three-dimensional systems to more faithfully mimic in vivo conditions [3]. Because patients react differently to diverse liver injuries, it is crucial that liver fibrosis models also recapitulate the variance of liver disease patient responses. Recently, there has been an increasing effort to model liver fibrosis with induced pluripotent stem cell (iPSC)-derived liver cells, expandable cells that can be derived from patients and differentiated towards different liver cells, reflecting their diverse genetic backgrounds. Although there have been major improvements in the differentiation of iPSCs towards both hepatocytes and HSCs, iPSC-derived hepatocytes (iHepatocytes) still do not fully recapitulate the metabolic functionality of primary hepatocytes, and iPSC-derived HSCs (iHSCs) express only a subset of primary HSC markers [4,5]. The potential of PSC-derived in vitro liver models for high-throughput drug screening has been demonstrated in organoid cultures [6]. However, the functionality of these models is still not at the level of human-donor-derived primary hepatocyte models, even though cell-type-specific gene expression levels seem similar [7]. Furthermore, fibrosis models consisting of both iPSC-derived hepatocytes and HSCs show high variability and activation due to suboptimal culture conditions [8].

Here, we report the identification of retinoid X receptor alpha (RXRA) as a transcription factor playing an important role in hepatocyte differentiation and HSC quiescence. The addition of its natural ligand 9-cis-retinoic acid (9CRA) to liver differentiation cultures improves the differentiation of stem-cell-derived hepatocytes and HSCs, resulting in a more physiologically relevant liver fibrosis model. 9CRA not only improves iHepatocyte functionality but also makes the iHSCs less activated, resulting in an improved capacity to model liver fibrosis and drug-induced liver injury.

## 2. Materials and Methods

Suppliers and catalogue numbers of all products and cell lines mentioned are listed in Table 1.

### 2.1. iPSC Culture Maintenance

Induced pluripotent stem cells (iPSCs) were kept in culture in Biolaminin-coated plates with Essential 8 flex medium. Biolaminin was diluted 1/10–1/20 in DPBS with calcium and magnesium. When a confluency of about 80% was reached, cells were detached with 1 mL of StemPro Accutase Cell Dissociation Reagent per well of a 6-well plate. Cells were incubated for 2 to 4 min, Accutase was aspirated, and cells were pipetted loose. Cells were counted, and 300,000 to 400,000 cells per well of a 6-well plate were seeded together with 1/100 RevitaCell Supplement 100X. The medium was changed every day, but weekends were skipped by adding double the amount of medium on the day before the weekend.

### 2.2. Differentiation of iPSCs Towards Hepatic Stellate Cells (iHSCs)

iPSCs were passaged and seeded as described before, with a density of 400,000 cells per well of a 6-well plate, but mTeSR1 complete medium was used instead of Essential 8 flex. The medium was changed with mTeSR1 until the cells reached a confluency of 60%. Then, liver differentiation medium (LDM) was used to start the differentiation, as described by Coll et al. [5]. The following factors were added to LDM: 50 ng/mL of BMP from day 0 to day 6, 20 ng/mL of aFGF from day 4 to day 8, and 5 µM of retinol and 100 µM of palmitic acid from day 6 to day 10. Cells were treated with 2 µM of 9-cis-retinoic acid (9CRA) or dimethyl sulfoxide (DMSO) as a control from day 6 of differentiation onwards, and the medium was changed every other day.

For the spheroid cultures, passaged iHSCs were used. For this, cells were passaged 1/3 on day 10 of differentiation using 1 mL of Trypsin-EDTA 0.05% per well of a 6-well plate. Cells were incubated for 8 min, scraped loose, and cultured in Dulbecco’s modified medium with 2.5% HEPES Buffer solution, 1% Penicillin–Streptomycin, and 10% Foetal Bovine Serum (DMEM 10%), to which 1/100 RevitaCell was added. When passage one iHSCs reached 90% confluency, they were frozen to be used later in spheroid cultures. To do so, they were detached again with Trypsin-EDTA 0.05%, centrifuged at 1750 RPM for 8 min, and frozen in DMEM with 10% DMSO and 20% FBS at a density of approximately two million cells/mL.

### 2.3. Differentiation of iPSCs Towards Hepatocytes (iHepatocytes)

iPSCs were genetically engineered to overexpress the transcription factors HNF1α, FOXA3, and PROX1 through induction of a Tet-ON system with doxycycline hyclate (DOX) [7]. As described for iHSC differentiation, iPSCs were seeded with mTeSR with a density of 400,000 cells per well of a 6-well plate or 20,000 cells per well of a 96-well plate. Then, liver differentiation medium (LDM) was used to start the differentiation, as described by Boon et al. [4]. The following factors were added to LDM: 6 µL/mL of DMSO (day 0 to 21), 50 ng/mL of Activin A (day 0 to 4), 50 ng/mL of Wnt3a (day 0 to 2), 5 µg/mL of DOX (day 4 to 21), 50 ng/mL of BMP4 (day 4 to 8), 20 ng/mL of aFGF (day 8 to 12), 20 ng/mL of HGF (day 12 to 21), 7.7% essential and 15.4% non-essential amino acids (day 12–21), and 2% L-Serine (day 14 to 21). The treatment with 2 µM of 9CRA or DMSO as a control was started from day 4 onwards, and the medium was changed every other day.

### 2.4. iPSC-Derived Liver Organoids

Thawed passage two iHSCs were used together with day eight iPSC-derived hepatoblasts (iHeps) to form spheroids. iHSCs were detached as described above, centrifuged at 1750 RPM for 8 min, and resuspended in seeding medium (LDM with 20 ng/mL of aFGF, 6 µL/mL of DMSO, 5 µg/mL of DOX, and 10% FBS). iHeps were incubated with 1 mL of Trypsin-EDTA 0.05% per well of a 6-well plate for 8 min, after which the cells were detached through pipetting. Both cell types were resuspended with seeding medium. Spheroids were seeded as described before by Cools et al. [7]. Briefly, a total of 2000 cells in a volume of 10 µL seeding medium with 1/100 RevitaCell and a ratio of 2:1 of iHSCs:iHepatoblasts was seeded per well of a 96-well ultra-low attachment U-bottom plate. After seeding, the plates were centrifuged at 800 RPM for 5 min and kept on an orbital shaker at 80 RPM at 37 °C. One day after seeding, 100 µL of fresh medium was added per well. From day 3 after seeding onwards, the medium was changed every other day for 13 days by taking off 100 µL of medium per well and adding 100 µL of fresh medium. The following components were added to the spheroid culture medium: 6 µL/mL of DMSO (day 1 to 13), 5 µg/mL of DOX (day 1 to 13), 20 ng/mL of aFGF (day 1 to 5), 20 ng/mL of HGF (day 5 to 13), 7.7% essential and 15.4% non-essential amino acids (day 5 to 13), and 2% L-Serine (day 7 to 13). Cells were treated with 2 µM of 9CRA or DMSO as a control from the day of seeding onwards, and the medium was changed every other day.

### 2.5. Isolation of pHSCs and Hepatocytes

Post-operative human liver pieces were temporarily stored in IGL-1 organ preservation solution for a maximum of 2 h until isolation of cells, as described by Cools et al. [7]. Liver pieces were perfused with three different solutions at a flow rate of 7.5 mL/minute. First, they were perfused with the first perfusion buffer (Table S1) for 5 min. This was followed by 5 min of perfusion with the second perfusion buffer (Table S1) containing 0.5 mg/mL of Pronase E and, lastly, another 5 min of perfusion with the second perfusion buffer supplemented with 0.25 mg/mL of Collagenase P. Then, the liver fragments were disrupted into small pieces using forceps, after which they were incubated in the second perfusion buffer containing 0.5 mg/mL of Pronase E and 0.5 mg/mL of Collagenase P at 37 °C for 20 min. At the 5 and 10 min marks, a drop of NaOH (pH 14) was added to the cell solution until a single-cell suspension was reached. This was passed through a metal sieve to remove debris and centrifuged at 50 g for 7 min to pellet the hepatocytes. The supernatant was centrifuged again, but this time at 1750 RPM for 8 min. The resulting cell pellet was incubated with red blood cell lysis solution for 3 min at room temperature, washed with the second perfusion buffer, and centrifuged at 1750 RPM for 8 min. The cell pellet was resuspended in an 18% Nycodenz solution with DNase I. This was consecutively topped off with 12% and 8.2% Nycodenz. Cells were centrifuged at 1450 g for 20 min, after which the HSCs between the Nycodenz gradients were collected and resuspended in PBS. After final centrifugation at 1750 RPM for 8 min, cells were resuspended in DMEM 20% that was switched to DMEM 10% from the next day onwards. In the meantime, the hepatocyte pellet was resuspended in the second perfusion buffer, and the cell suspension was pipetted onto a Percoll gradient. After centrifugation at 50 g for 20 min, the cell pellet was resuspended in ice-cold PBS and incubated for 3 min with red blood cell lysis at room temperature.

### 2.6. pHSC/HepaRG Spheroids

HepaRGs (Biopredic) were thawed and resuspended in thaw/seed medium HTM (Williams E medium, 1% Glutamax supplement, and ADD670 supplement) and centrifuged at 500× *g* for 3 min. Cells were counted with trypan blue. Passage three or four pHSCs cultured in DMEM 10% FBS were detached as described above for iHSCs, centrifuged at 1750 RPM for 8 min, resuspended in HTM, and counted. Per spheroid, a total of 2000 cells with a 2:1 ratio of pHSC:HepaRG was seeded in 10 µL of HTM [9]. Then, 24 h later, 100 µL of culture medium HC0DM (Williams E medium, 1% Glutamax supplement, and ADD610) was added to each well, and the medium was changed one more time with HC0DM before switching to HFM maintenance medium (Williams E medium, 1% Ultraglutamine, ADD650, 0.5% DMSO, 10 ng/mL of HGF, and 2 ng/mL of EGF) on day four of the 3D culture. From this point, the medium was changed every other day for 14 days by removing 100 µL of medium and adding 100 µL of fresh HFM medium.

### 2.7. Organoid Exposure to TGFβ and APAP

To compare the induction of iHSC activation in 9CRA-treated spheroids versus control spheroids, they were exposed to 25 ng/mL of transforming growth factor beta (TGFβ) for 48 h to directly induce iHSC activation. Once exposure to TGFβ was started, the treatment with 9CRA was stopped. Six spheroids per condition were lysed for RT-qPCR analysis, and six spheroids were fixed to perform Picrosirius staining.

To assess iHepatocyte functionality in the spheroids and the capacity of the iHSCs to activate through hepatocyte damage, spheroids were exposed to different concentrations of acetaminophen (APAP), including 0, 5, 10, 15, 20, 30, 40, 60, 80, and 100 mM for 72 h. Once the exposure to APAP was started, the treatment with 9CRA was stopped. Six spheroids per condition were lysed for RT-qPCR analysis, and six spheroids were fixed to perform Picrosirius staining.

### 2.8. Cell Viability Assay

IC50 of APAP in spheroids was determined through ATP measurement with the CellTiter-Glo Luminescent Cell Viability assay. In short, spheroids were treated with different concentrations of APAP ranging from 0 to 100 mM. Single spheroids in 100 µL of medium each were transferred to black bottom 96-well plates. Then, to each well, 100 µL of the assay substrate/enzyme mix was added. Spheroids were resuspended, incubated on a shaker for 10 min, and incubated off of the shaker for another 10 min at room temperature. Luminescence was measured with a GloMax (Promega).

### 2.9. RNA Extraction and RT-qPCR

For 2D differentiated cells, either 1 well of a 12-well plate or 3 wells of a 96-well plate were lysed, and RNA was extracted with the ReliaPrep RNA Cell Miniprep System. RNA concentrations were measured with the Nanodrop 2000 Spectrophotometer, and all samples were diluted to the same concentration. For spheroids, 6 spheroids per condition were used, and the same procedure was followed to retrieve RNA. RNA was reverse transcribed into cDNA with M-MLV Reverse Transcriptase, RNase H Minus, and Point Mutant, following the manufacturer’s protocol. qPCR was performed with the QuantStudio 3 Real-Time PCR System using the GoTaq qPCR Master Mix with BRYT Green Dye according to the manufacturer’s protocol. GAPDH and RPL19 were used as reference genes, and primers specific to the following genes of interest were used: ACTA2, COL1A1, LOX2, LRAT, DCN, CYP3A4, CYP2C9, CYP1A2, and HNF4α. An overview of all primer sequences is given in Table 1.

### 2.10. Immunofluorescence Stainings

Differentiated cells in 2D were fixed in 12- or 96-well plates through incubation with 4% formaldehyde solution for 10 min at room temperature. Cells were permeabilised with 0.1% Triton-X100 for 5 min three times, blocked with 2% bovine serum albumin (BSA) dissolved in PBS for 30 min, and incubated overnight at 4 °C with the primary antibody diluted in 0.1% Triton-X100 and 1% BSA. Next, cells were again washed for 5 min three times with 0.1% Triton-X100 and incubated for 2 h at room temperature with the secondary antibody diluted in 0.1% Triton-X100 and 1% BSA. Then, cells were washed three times for 5 min with PBS and one time with distilled water before mounting with a Fluorescence Mounting Medium to which DAPI dilactate was added with a dilution of 1/5000. Images were taken with an EVOS. An overview of all antibodies is given in Table 1.

### 2.11. Picrosirius Staining

Spheroids were fixed with 4% formaldehyde solution for 10 min. Then, they were embedded in paraffin and sliced into 5 µm thin sections that were mounted onto microscope slides. The sections were deparaffinised for 10 and then 5 min in xylene and rehydrated with decreasing concentrations of 2-Propanol (two times 100%, 90%, and 70%) before 10 min of incubation at room temperature with methanol. Slides were then incubated with a mix of Sirius Red and Fast Green FCF, dissolved in a picric acid solution for 1 h at room temperature, washed with distilled water, and dehydrated again in 2-Propanol with increasing concentrations (70%, 90%, and 100% two times). Lastly, slides were mounted with DPX mounting medium for histology and imaged with an EVOS.

### 2.12. Flow Cytometry Analysis

One well of a six-well plate per condition of iHSCs on day 10 of differentiation was incubated with Trypsin-EDTA 0.05% for 8 min at 37 °C, after which the cells were scraped loose and filtered through a 40 µm cell strainer to obtain a single-cell suspension. Cells were centrifuged at 1750 RPM for 8 min and resuspended with 1 mL of PBS per condition. To distinguish living cells from dead cells, fixable viability stain 780 was used with a 1/1000 dilution. Cells were incubated with this dye for 15 min at room temperature, and then 4 mL of 1% BSA in PBS was added to each tube. Cells were centrifuged again at 1750 RPM for 8 min. Afterwards, they were fixed with 4% formaldehyde solution for 10 min at room temperature and washed with PBS.

To investigate the change in vitamin A storage after treatment with 9CRA, flow cytometry analysis was performed on a FACS Aria III. Analysis was performed based on retinyl esters that have an excitation wavelength of 355 nm and an emission wavelength of 450 nm. As a negative control, cells that were not exposed to retinol or 9CRA during differentiation and not stained with the viability stain were used. As a positive control for the viability stain, cells were killed by microwaving them for 10 s. The gating strategy is displayed in Figure S1.

### 2.13. CYP3A4 Activity Assay

iPSCs were differentiated towards iHepatocytes in 96-well plates as described before, and one part of the cells was treated with 9CRA from day 4 of differentiation onwards. Because DMSO and 9CRA can inhibit CYP3A4 activity, 72 h prior to P450-Glo CYP3A4 activity assay performance on day 21 of differentiation, DMSO and 9CRA were left out of the medium. The assay was performed according to the manufacturer’s instructions. In short, cells were washed with PBS and exposed to the luminogenic substrate, Luciferin-IPA, for one hour at 37 °C. Subsequently, the medium of the cells was transferred to an opaque, white, 96-well plate to which the Luciferin Detection Reagent was added and incubated for 20 min at room temperature. Luminescence was measured with a Glomax. The background of empty wells was subtracted from the measured luminescence. Values of treated cells were then divided by the values of the control conditions and multiplied by 100 to calculate the percentage change.

### 2.14. RNA Sequencing and Analysis

RNA of freshly isolated pHSCs and PHHs, freshly thawed HepaRGs, iPSCs, and iHepatoblasts and iHepatocytes differentiated with or without 9CRA was extracted as described before. The quality of the RNA was determined with a Bioanalyzer 6000 to make sure all samples were of sufficient quality and had a concentration higher than 10 ng/µL. Library preparation was performed with the QuantSeq 3′ mRNA-Seq V2 Library Prep Kit FWD with UDI 12 nt set B1. The libraries were sequenced with NovaSeq SP 100x6bp. Quality control of sequencing reads was conducted using FastQC, followed by adapter trimming and error correction with BBDuk [10]. The processed reads were aligned to the reference genome (Homo Sapiens_GRCm38.p6) using the STAR aligner [11]. Transcript assembly was carried out with HTSeq [12]. Raw count matrices were generated and normalised with the DESeq2 package in R [13]. The clusterProfiler package was used for over-representation analysis of differentially expressed genes [14]. Gene set variation analysis (GSVA) of Reactome gene sets was performed with the GSVA package in R [15]. UMAP Visualisation of publicly available single-cell RNA sequencing datasets was performed using Seurat [16]. Datasets used include an HSC activation atlas consisting of isolated HSCs during different murine liver injury models and a dataset describing the maturation of murine hepatocytes in several embryonal and postnatal stages [17,18]. Inference of transcription factor activity and their downstream targets was carried out with the pySCENIC package in Python [19].

### 2.15. Statistical Analysis

Statistical analyses were conducted using Graphpad Prism 10.3.1. The Shapiro–Wilk normality test was performed when sample sizes were *n* ≥ 3. For qPCR fold change comparisons between control and 9CRA-treated cells or organoids, a one-tailed paired *t*-test was performed. Also, for the percentage of vitamin-A-positive cells and the quantification of the PSR-positive area after TGFβ or APAP exposure, a one-tailed paired *t*-test was carried out. A one-tailed unpaired *t*-test was used for selected genes of the bulk RNA sequencing to compare primary with iPSC-derived HSCs. For the PSR-positive area of day 13 control or 9CRA organoids, a one-tailed unpaired *t*-test was conducted. For qPCR fold changes or GSVA scores comparing more than two groups, an ordinary one-way ANOVA with Tukey’s multiple comparisons test was performed. Lastly, for the time course comparisons of qPCR ΔCt values of more than two groups, a two-way ANOVA with Tukey’s multiple comparisons test was conducted. Error bars represent the standard error of the mean. Other statistical details of the experiments can be found in the figures’ legends.

## 3. Results

### 3.1. iPSC-Derived Liver Organoids Are More Fibrotic than HepaRG/pHSC Organoids

**Figure 1:**
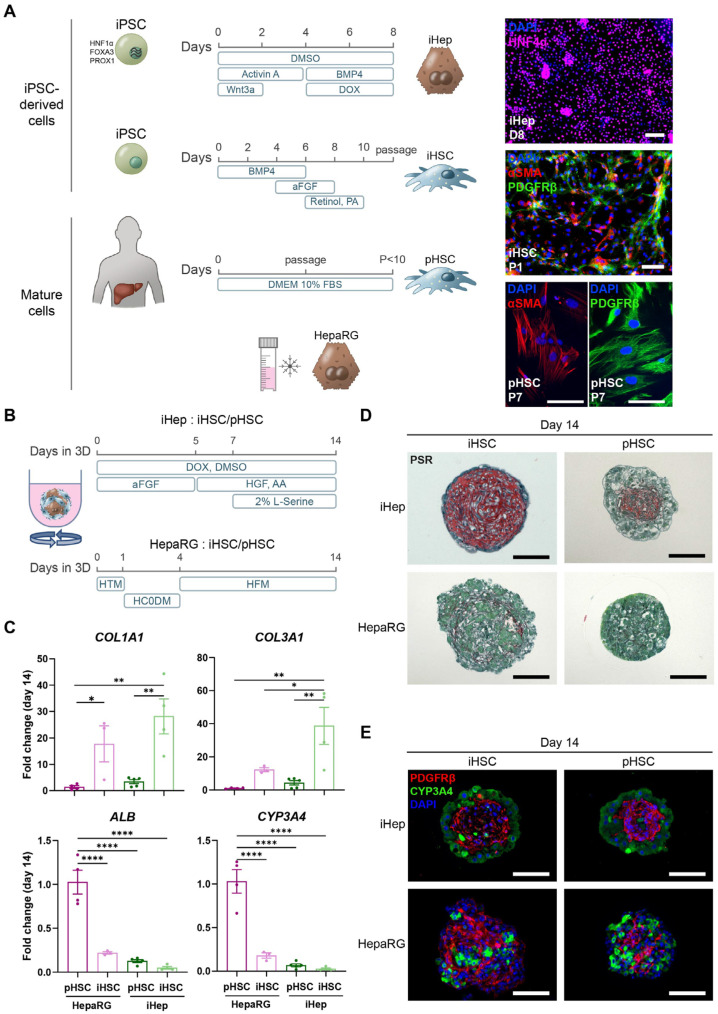
iPSC-derived liver organoids are more fibrotic than HepaRG/pHSC organoids. (**A**). Schematic overview of iPSC differentiations towards iHeps and iHSCs and 2D culture conditions of pHSCs and HepaRGs (left panel). Expression of cell-type-specific proteins (right panel). Further characterisation of iHSCs and iHepatocytes can be found in Figure S1. (**B**). Schematic overview of organoid culture conditions. iHeps were combined with passaged iHSCs or pHSC and cultured with iHepatocyte differentiation medium. HepaRGs, combined with iHSCs or pHSCs, were cultured with HepaRG culturing medium. (**C**). Gene expression levels of HSC activation markers *COL1A1* and *COL3A1* and hepatocyte markers *ALB* and *CYP3A4* on day 14 of the organoid culture. Fold changes were calculated using the HepaRG-pHSC spheroids as a control. Data are represented as mean ± SEM. *n* = 3–5, with 6 spheroids per repeat, * *p* < 0.05, ** *p* < 0.01, **** *p* < 0.0001. An overview of the expression of these genes over time in 3D can be found in Figure S2. (**D**). PSR staining, showing cross-linked collagen accumulation in spheroids on day 14 of the co-cultures. (**E**). Protein expression of the HSC marker PDGFRβ and the hepatocyte marker CYP3A4 on day 14 of the organoid cultures. Scale bars represent 100 µm, except for pHSC staining in panel A, where they represent 200 µm.

To model liver fibrosis with induced pluripotent stem cell (iPSC)-derived liver spheroid co-cultures composed of hepatocytes (iHepatocytes) and hepatic stellate cells (iHSCs), it is essential that iHSC activation can be induced and that metabolic functionality of iHepatocytes is sufficient to metabolise drugs. To assess whether these criteria were met in the iPSC-derived liver organoids, they were compared to liver organoids that contained their more mature counterparts: HepaRGs and primary human HSCs [9]. HepaRG cells are derived from a hepatocellular carcinoma and, when differentiated towards hepatocytes, closely mimic the functional characteristics of primary human hepatocytes [20,21]. Primary HSCs (pHSCs) were derived from liver resections and passaged at least three times [22]. iPSCs were differentiated towards HNF4α-positive hepatoblasts (iHeps) and PDGFRβ/αSMA-positive iHSCs (Figure 1A), following previously established differentiation protocols (Figure S1) [4,5]. For their more mature counterpart, PDGFRβ/αSMA-positive passaged pHSCs and freshly thawed differentiated HepaRGs were used (Figure 1A). These cells were integrated into free-floating liver organoids and cultured for 14 days, as previously described [7]. iHeps and HepaRGs were combined with either iHSCs or pHSCs to assess the functional contributions of iPSC-derived hepatocytes and HSCs versus their more mature counterparts. For the organoids containing iHeps, hepatocyte differentiation was continued, while the HepaRG organoids were cultured with HepaRG medium (Figure 1B).

Because it has been shown before that HSCs become more quiescent when co-cultured in organoids with hepatocytes [5], we investigated whether there would be a difference between iHSC and pHSC inactivation and whether the choice of hepatocyte used in the co-culture would have an influence on this process. Although starting levels of *COL1A1* gene expression in the cultures did not differ significantly, the *COL3A1* expression levels of organoids with iHSCs were significantly higher than those with pHSCs (Figure S2). This trend became clearer over time, showing the lowest level of HSC activation in the presence of pHSCs and the highest in the presence of iHSCs (Figure 1C and Figure S2). Compared to HepaRGs, the use of iHeps led to a higher HSC activation level by the end of the culture, regardless of the source of the HSCs (Figure 1C and Figure S2). Overall, the decrease over time of both *COL1A1* and *COL3A1* gene expression is the smallest in the iHep/iHSC spheroids, suggesting that in iPSC-derived organoids, HSCs do not inactivate as well as in HepaRG/pHSC cultures. To determine hepatocyte maturity, expression of hepatocyte markers *CYP3A4* and *ALB* was measured, showing a clear increase over time with all combinations (Figure S2). By day 14 of culture, the highest *CYP3A4* and *ALB* levels were reached in the HepaRG/pHSC spheroids, while the lowest levels were measured in the iHep/iHSC spheroids. While pHSCs effectively maintained the state of HepaRGs, they did not enhance the maturity of iHepatocytes, suggesting that iHepatocytes lacked the appropriate mechanisms to receive the signals from the pHSCs (Figure 1C and Figure S2). iHSCs, on the other hand, were unable to maintain the state of either HepaRGs or iHepatocytes, indicating that iHSCs themselves lacked the necessary signalling to promote or maintain hepatocyte maturity (Figure 1C and Figure S2). Overall, these data indicate that iHepatocyte and iHSC differentiations are suboptimal, both of which influence the activation status of the HSCs in co-cultures.

The higher activation in the iPSC-derived organoids was confirmed at the protein level with Picrosirius staining. iPSC-derived organoids produced a large amount of cross-linked collagen that was almost absent in the HepaRG/pHSC cultures (Figure 1D). Replacing either iHepatocytes or iHSCs with their more mature counterpart reduced the amount of cross-linked collagen inside of the organoids, confirming that the differentiation of both hepatocytes and HSCs is suboptimal in iPSC-derived liver organoids (Figure 1D). The lower levels of cross-linked collagen were not due to the absence of PDGFRβ-positive HSCs in the organoids, as all cultures had similar amounts of HSCs (Figure 1E).

### 3.2. Immaturity of iPSC-Derived Liver Cells

**Figure 2:**
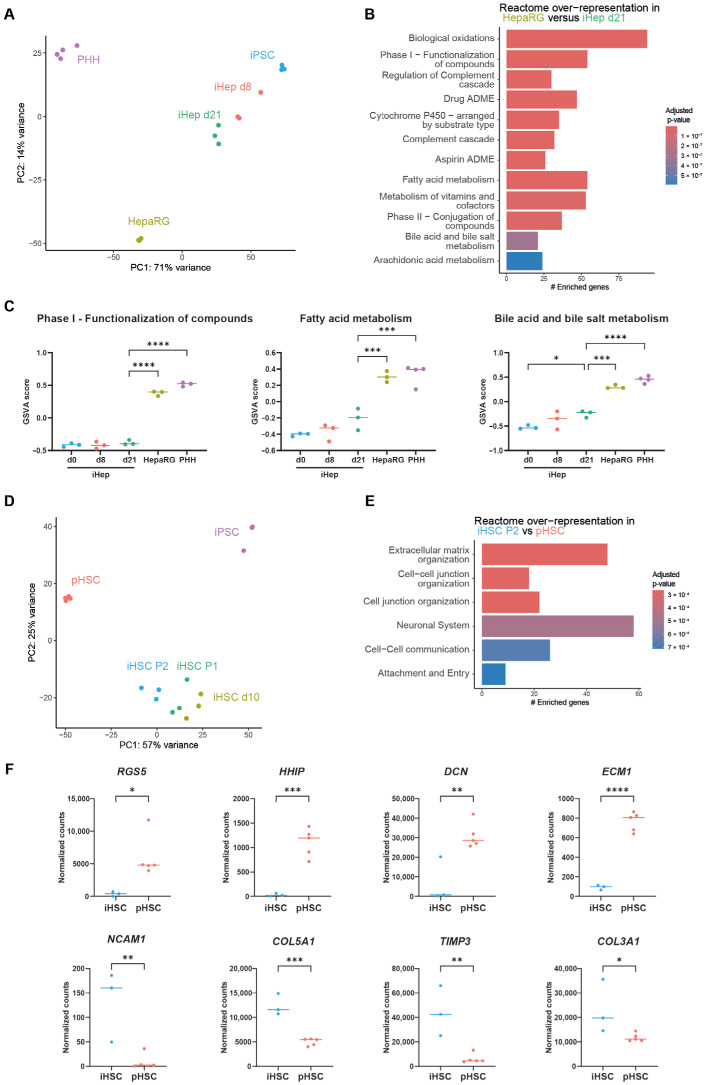
Immaturity of iPSC-derived liver cells. (**A**). Principal component analysis (PCA) of the transcriptomes of iHepatocytes (iHeps in figure) at different times during differentiation as well as HepaRGs and freshly isolated PHHs. (**B**). Pathways that are over-represented in the upregulated genes of HepaRGs compared to iHepatocytes at day 21 of differentiation (# Enriched genes). (**C**). Gene set variation analysis (GSVA) score for metabolic pathways in HepaRGs and PHHs and in iHepatocytes at different times during differentiation. *n* = 3–4, * *p* < 0.05, *** *p* < 0.001, **** *p* < 0.0001. Also, see Figure S3. (**D**). PCA of the transcriptomes of passaged primary HSCs and iHSCs at different times during differentiation. (**E**). Pathways that are over-represented in the upregulated genes in iHSCs after 2 passages compared to primary passaged HSCs. (**F**). Gene expression levels of quiescent and activated HSC markers in iHSCs and primary passaged HSCs. *n* = 3–5, * *p* < 0.05, ** *p* < 0.01, *** *p* < 0.001, **** *p* < 0.0001.

Even though PSC differentiations towards liver cells have greatly improved [23] and can be used in models of liver disease, so far, iPSC-derived liver cell types are still lacking some functionality when compared to their adult liver cell counterparts [24]. To investigate what we lack in iPSC-derived liver cells, we profiled the RNA expression of iPSCs directed towards hepatocytes and stellate cells and compared this with HepaRGs and primary human hepatocytes (PHHs) and stellate cells (pHSC). Bulk RNA sequencing confirms that the iPSC differentiations are not generating fully differentiated liver cells (Figure 2A). Compared to more mature hepatocytes (HepaRGs), iHepatocytes had significantly lower expression of genes in pathways related to the metabolic activity of mature hepatocytes (Figure 2B). More specifically, drug metabolism, fatty acid metabolism, and bile acid metabolism pathways did not reach mature hepatocyte levels at the end of iHepatocyte differentiation (Figure 2C), which was also demonstrated by the low expression values of *CYP3A4*, *CYP2C9*, *NR1H4*, *ABCB11*, *EHHADH*, and *HADH* (Figure S3).

Similarly, iPSC-derived HSCs show a different expression pattern than mature primary human HSCs (Figure 2D). Compared to pHSCs, iHSCs show high expression of genes that are associated with extracellular matrix organisation, indicating that iHSCs have a more activated phenotype than pHSCs (Figure 2E). Notably, iHSCs have lower expression of the quiescent HSC genes *DCN*, *ECM1*, *HHIP*, and *RGS5*, which inhibit TGF-β and Hedgehog pathways as well as G-protein signalling (Figure 2F) [25,26,27,28]. In addition, iHSCs have higher expression of extracellular matrix and cellular adhesion related genes associated with HSC activation (Figure 2F). Together, these results indicate that iHepatocytes are metabolically immature and that iHSCs are more fibrotic than the donor-derived liver cells.

### 3.3. RXRA as a Target for Improving iPSC-Derived Liver Cells

**Figure 3:**
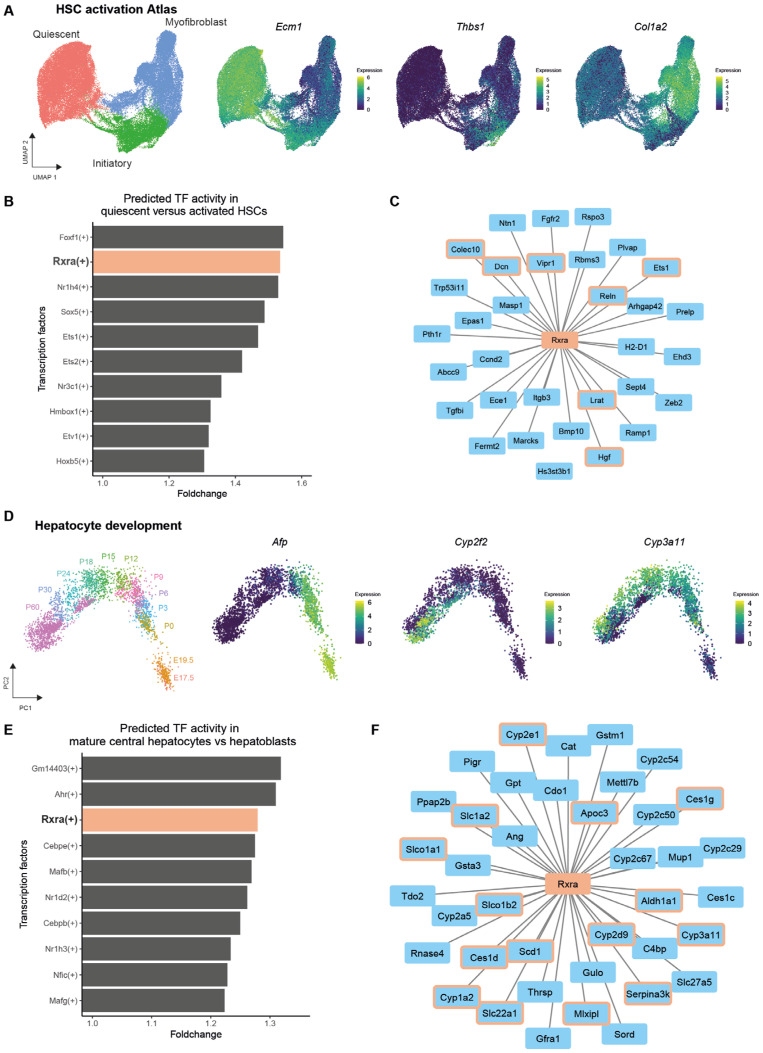
RXRA as a target for improving iPSC-derived liver cells. (**A**). Uniform Manifold Approximation and Projection (UMAP) of the HSC activation atlas with normalised expression of canonical markers of distinct stages of HSC activation. (**B**). Fold change in TF activity in quiescent HSCs compared to activated HSCs (myofibroblasts). The top 10 TF activities inferred with pySCENIC in quiescent HSCs are visualised. (**C**). Downstream target genes of RXRA in HSCs as predicted by pySCENIC. Canonical quiescent HSC markers are indicated with an orange border. Also, see Figure S4. (**D**). PCA of hepatocyte development from hepatoblasts at embryonic day (**E**) 17.5 to mature central and portal hepatocytes at day 60 postnatally (P) with canonical markers at the different stages [18]. (**E**). Fold change in TF activity in mature central Heps compared to hepatoblasts. The top 10 TF activities inferred with pySCENIC in mature central Heps are visualised. (**F**). Downstream target genes of RXRA in Heps as predicted by pySCENIC. Canonical markers for Hepatocyte metabolism are indicated with an orange border.

In order to identify key transcription factors that could steer the differentiation of both iHepatocytes and iHSCs, we examined single-cell RNA sequencing (scRNAseq) data from quiescent and activated HSCs, as well as immature and mature hepatocytes from mice. The HSC activation atlas depicts the transition from quiescent HSCs (*Ecm1*) to initiatory HSCs (*Thbs1*) and, finally, to myofibroblasts (*Col1a2*) from 10 different scRNAseq datasets described in Merens et al. (Figure 3A) [17]. Gene regulatory network inference with pySCENIC identified Forkhead Box F1 (FOXF1) as the transcription factor with the highest activity in quiescent HSCs compared to activated HSCs (Figure 3B). However, FOXF1 lacks a natural ligand. In contrast, the nuclear receptor Retinoid X Receptor Alpha (RXRA), which ranked second in activity, does have a natural ligand: 9-cis-retinoic acid (9CRA). Predicted downstream target genes of RXRA in quiescent HSCs include *Dcn*, *Lrat*, *Hgf*, *Reln*, *Ets1*, *Vipr1*, and *Colec10* (Figure 3C). The high activity of RXRA in quiescent HSCs is unique among mesenchymal cells; fibroblast-like cells from other organs or non-HSC mesenchymal cells in the liver have distinctly lower inferred TF activity of RXRA compared to HSCs (Figure S4). To identify TFs that are responsible for the greater metabolic function of mature hepatocytes compared to hepatoblasts, we used single-cell RNA sequencing data describing hepatocyte development from hepatoblasts (*Afp+*) at embryonic day 17.5 to mature central (*Cyp3a11+*) and portal (*Cyp2f2+*) hepatocytes at day 60 postnatally (Figure 3D) [18]. Gene regulatory network inference with pySCENIC showed that RXRA is not only highly active in quiescent HSCs compared to activated HSCs but also in mature central hepatocytes compared to hepatoblasts (Figure 3E). Predicted downstream target genes of RXRA in mature central hepatocytes include *Cyp2e1*, *Cyp3a11*, *Cyp1a2*, *Cyp2d9*, *Slc01a1*, *Slc1a2*, and *Mlxipl* (Figure 3F). Collectively, these results suggest that inducing RXRA could enhance the maturation of iPSC-derived liver cells.

### 3.4. 9-cis-Retinoic Acid Treatment Improves HSC Differentiation from iPSCs

**Figure 4:**
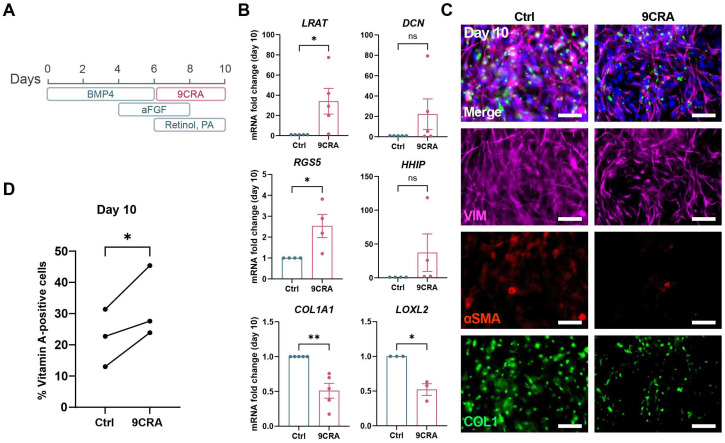
9-cis-retinoic acid treatment improves HSC differentiation from iPSCs. (**A**). Schematic representation of the differentiation of iPSCs towards iHSCs with the addition of 2 µM of 9CRA from day 6 to day 10 of differentiation. (**B**). Gene expression levels of the quiescent HSC genes *LRAT*, *DCN*, *RGS5*, and *HHIP* and HSC activation genes *COL1A1* and *LOXL2* on day 10 of iHSC differentiation with or without 9CRA treatment. Data are represented as mean ± SEM. *n* = 3–5, ns *p* > 0.05, * *p* < 0.05, ** *p* < 0.01. (**C**). Protein expression of VIM, αSMA, and COL1 on day 10 of iHSC differentiation with or without 9CRA treatment. Scale bars represent 50 µm. (**D**). Vitamin A uptake in day 10 iHSCs differentiated with or without 9CRA determined through flow cytometry analysis. Also, see Figure S5 for flow cytometry gating strategy (* *p* < 0.05).

To investigate the potential effect of RXRA on iHSC differentiation, cells were treated with 9CRA, an RXRA agonist, from day 6 of the differentiation onwards (Figure 4A). This led to significantly higher gene expression levels of quiescent HSC markers *LRAT* and *RGS5* and slightly higher expression levels of *DCN* and *HHIP* (Figure 4B). In contrast, activation genes *COL1A1* and *LOXL2* were significantly downregulated by the end of differentiation (Figure 4B). Moreover, there seemed to be less αSMA- and COL1-positive iHSCs after treatment with 9CRA (Figure 4C). One of the functions of quiescent HSCs is the storage of vitamin A or retinol in lipid droplets. This process is facilitated by LRAT, which is an enzyme that esterifies retinol into retinyl esters [29]. Because 9CRA led to increased *LRAT* gene expression, we investigated whether it could also improve vitamin A storage. Flow cytometry analysis showed a significantly higher uptake of retinol after treatment with 9CRA (Figure 4D and Figure S5). This suggests that 9CRA treatment of iPSC cultures differentiating towards iHSCs generates less activated and more functional iHSCs.

### 3.5. 9-cis-Retinoic Acid Makes iHepatocytes More Mature

**Figure 5:**
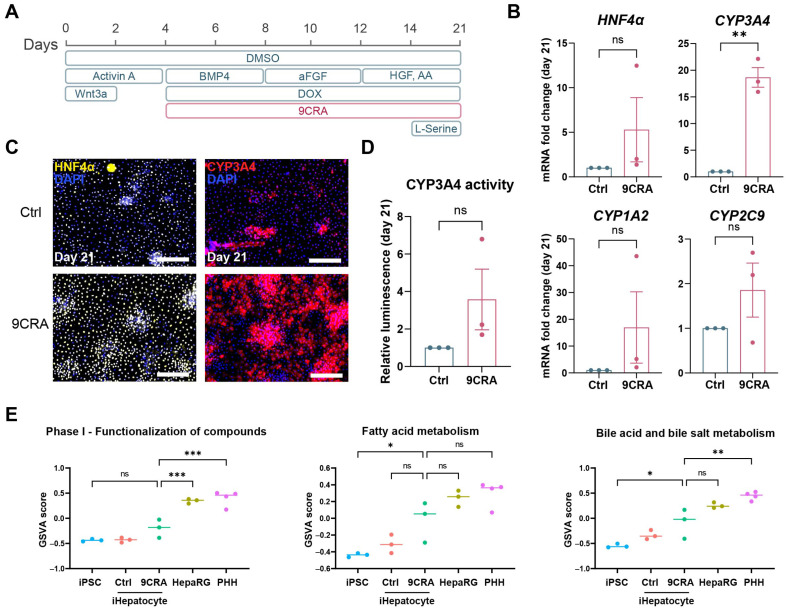
9-cis-retinoic acid makes iHepatocytes more mature. (**A**). Schematic representation of the iHepatocyte differentiation protocol with the addition of 2 µM of 9CRA from day 4 to day 21. (**B**). Gene expression levels of the master transcriptional regulator *HNF4α* and the mature hepatocyte genes *CYP3A4*, *CYP1A2*, and *CYP2C9* of day 21 iHepatocytes differentiated with or without 9CRA. Data are represented as mean ± SEM. *n* = 3, ns *p* > 0.05, ** *p* < 0.01. (**C**). Expression of the hepatocyte proteins HNF4α and CYP3A4 in day 21 iHepatocytes differentiated with or without 9CRA. Scale bars represent 200 µm. (**D**). CYP3A4 activity levels in day 21 iHepatocytes differentiated with 9CRA compared to those differentiated without (*n* = 3). Data are represented as mean ± SEM. (**E**). GSVA score for metabolic pathways in iPSCs, iHepatocytes (+/− 9CRA), HepaRGs, and PHHs. *n* = 3–4, ns *p* > 0.05, * *p* < 0.05, ** *p* < 0.01 *** *p* < 0.001. Also see Figure S6.

To evaluate the impact of 9CRA treatment on the differentiation of iPSCs towards iHepatocytes, cells were treated with 9CRA from day 4 of differentiation onwards (Figure 5A). This led to a significant upregulation of *CYP3A4* and elevated trends in the expression of CYP1A2, CYP2C9, and the master transcriptional regulator of hepatic function, *HNF4α* [30] (Figure 5B and Figure S6). Immunofluorescent staining also showed a higher number of HNF4α- and CYP3A4-positive cells after treatment with 9CRA (Figure 5C). Furthermore, an increased tendency of CYP3A4 activity levels was observed in the cells treated with 9CRA (Figure 5D). RNA sequencing of 9CRA-treated cultures showed that while 9CRA increased the expression of genes related to bile, fatty acid, and drug metabolism, 9CRA-treated iHepatocytes did not reach the expression levels of mature hepatocytes (Figure 5E). We also tested 9CRA treatment from day 8 onwards, which similarly resulted in increased *CYP3A4* expression by day 21 (Figure S7). However, because treatment from day 4 onwards already improved *CYP3A4* expression at day 8 of differentiation, we opted for this treatment protocol. Taken together, these data demonstrate that the addition of 9CRA to the differentiation medium of iHepatocytes enhances the expression of crucial hepatocyte genes and consequently augments their functionality.

### 3.6. 9-cis-Retinoic Acid Improves iPSC-Derived Liver Fibrosis Modelling

**Figure 6:**
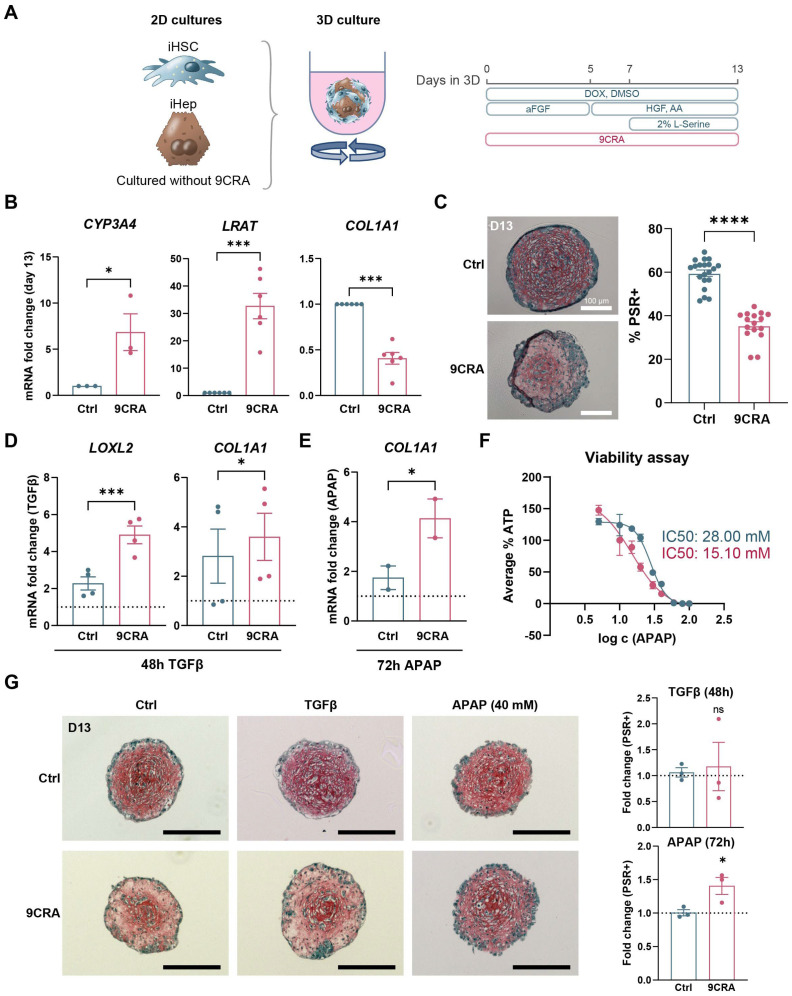
9CRA improves iPSC-derived liver fibrosis modelling. (**A**). Schematic representation of the spheroid cultures with the addition of 2 µM of 9CRA during the entire 3D culture. (**B**). Gene expression levels of *CYP3A4*, *LRAT*, and *COL1A1* at the end of the organoid culture with or without the addition of 9CRA. *n*= 3–5, with 6 spheroids per repeat, * *p* ≤ 0.05, *** *p* < 0.001. (**C**). PSR staining on day 13 of organoid culture with quantification of the percentage of cross-linked collagen-positive area in organoids cultured with or without 9CRA. Scale bar represents 100 µm, **** *p* < 0.0001. (**D**). mRNA levels of *LOXL2* and *COL1A1* on day 13 after 48 h of exposure to 25 ng/mL of TGFβ. *n* = 4, with 6 spheroids per repeat, * *p* ≤ 0.05, *** *p* < 0.001. (**E**). mRNA levels of *COL1A1* in organoids after 72 h of exposure to 40 mM of the hepatotoxic compound APAP. *n* = 2, with 6 organoids per repeat, * *p* < 0.05. (**F**). ATP viability assay performed on organoids with or without 9CRA supplementation of the culture and 72 h of exposure to different concentrations of APAP. Amount of ATP present in organoids relative to the untreated control. (**G**). PSR staining on control organoids and organoids exposed to TGFβ or APAP, with quantification of the percentage of cross-linked collagen-positive area in the organoids. Scale bar represents 200 µm, ns *p* > 0.05, * *p* < 0.05. All data are represented as mean ± SEM. Also, see Figure S7.

Because 9CRA improved iHepatocyte and iHSC cultures in 2D differentiations, we next investigated whether iPSC-derived liver organoids could also be improved by continuing 9CRA treatment in 3D. 9CRA-treated passaged iHSCs and iHeps were combined into spheroids with a ratio of 2:1 using an orbital shaker and 96-well cell-repellent U-bottom plates, as described previously [7]. Because immature hepatoblasts were integrated into the spheroid co-cultures, the hepatocyte differentiation protocol was continued in 3D. Additionally, 9CRA was added to the medium throughout the entire 3D culture period, which was 13 days (Figure S8A). This resulted in considerably smaller spheroids that were much more difficult to handle (Figure S8C). For this reason, we investigated whether treating the cells in 3D alone could also be beneficial for the liver organoids (Figure 6A). Similarly to the results observed in the 2D differentiations and organoids consisting of these cells, this led to a significant increase in gene expression of the mature hepatocyte gene *CYP3A4* and the quiescent HSC gene *LRAT* and significantly lower expression of the HSC activation gene *COL1A1* (Figure 6B and Figure S8B). Moreover, the decrease in *COL1A1* due to the 9CRA treatment was translated into a significantly lower amount of cross-linked collagen in the liver organoids (Figure 6C and Figure S8C). To determine whether this would lead to better detection of induction of iHSC activation, we exposed the liver organoids to transforming growth factor beta (TGFβ). TGFβ exposure of 9CRA organoids resulted in significantly higher induction of activation genes *LOXL2* and *COL1A1* (Figure 6D). Moreover, indirect induction of iHSC activation through iHepatocyte damage by the hepatotoxic compound acetaminophen (APAP, 40 mM) was also improved (Figure 6E). It is most likely that this was not only due to better functionality of the iHSCs but also the enhanced drug metabolism capacity of the iHepatocytes, as an IC50 of 15.10 mM for APAP was measured in the 9CRA-treated organoids compared to 28 mM in the control organoids (Figure 6F). Quantifications of picrosirius staining showed an increase in the amount of cross-linked collagen in the 9CRA-treated organoids after exposure to APAP, which could not be observed in the untreated organoids (Figure 6G). In summary, 9CRA treatment resulted in more functional iHSCs with reduced basal activation levels and more mature and functional iHepatocytes in the iPSC-derived liver organoids, enabling more advanced drug-induced liver fibrosis modelling.

## 4. Discussion

Since their discovery in 2006, iPSCs have gained popularity in the field of in vitro disease modelling, including chronic liver disease [31]. Although there have been major advances thus far in the development of iPSC-derived liver co-cultures, these models generally still show foetal characteristics, making them not yet sufficiently reliable for screening unknown compounds without additional independent validation [32]. Therefore, it is important to invest in the improvement of differentiation protocols and the final liver cultures.

Here, we compare iPSC-derived liver organoids with mature primary liver organoids. We confirm that iPSC-derived liver cells can be further optimised and identify RXRA as a key transcription factor regulating hepatocyte metabolism and HSC quiescence. We demonstrate the benefits of the RXRA ligand 9CRA in hepatic differentiation in both 2D mono- and 3D co-cultures. 9CRA not only reduced HSC activation but also improved hepatocyte maturation, leading to improved iPSC-derived in vitro liver disease modelling.

9CRA is a vitamin A metabolite that binds to nuclear retinoid X receptor (RXR). RXR can form homodimers or heterodimers with potentially more than 20 other nuclear receptors, including retinoic acid (RAR), thyroid hormone, and peroxisome proliferator-activated receptors (PPARs) [33,34]. After dimerization, RXR dimers can bind to DNA at hormone response elements or retinoid X response elements. In the absence of the ligand, the RXR dimer recruits corepressors that inhibit transcription. Upon ligand binding, the RXR dimer recruits coactivators, leading to the initiation of transcription [35]. Here, we inferred that RXRA has the potential to induce genes crucial for hepatocyte metabolism as well as HSC quiescence, which could be verified with functional assays.

We mainly focused on the drug metabolism capacity of the hepatocytes and drug-induced liver fibrosis, but, based on RNA sequencing data, we could conclude that 9CRA also improved fat and bile acid metabolism in iHepatocytes differentiated in 2D (Figure 5E). This suggests that 9CRA has the potential to also improve modelling of other chronic liver diseases, like metabolic dysfunction-associated steatotic liver disease (MASLD) and cholestasis. In addition to modelling liver diseases, improved iHepatocyte differentiation protocols offer opportunities for direct therapeutic applications in hepatic disorders as a renewable cell source for treating acute liver failure or inherited liver diseases, reducing the reliance on scarce donor livers [36,37]. Although HepaRGs and pHSCs are more mature than iPSC-derived liver cells, iPSC-derived liver cells could be more relevant as therapeutics due to their ability to circumvent the immune system as they can be made from the patient’s own cells. The therapeutic use of iPSC-derived cells has recently been demonstrated for the transplantation of iPSC-derived pancreatic cells to combat type 1 diabetes (NCT03163511) [38]. The ability to create better iPSC-derived liver cells will undoubtedly lead to better liver disease models and therapeutics in the future.

Interestingly, evidence for the presence of 9CRA in the liver is scarce. In 1992, it was detected for the first time in the liver and the kidney [39], but these results could not be reproduced for several years [40]. Only in 2023 was robust evidence obtained demonstrating the wide distribution of 9CRA across several tissues, including the liver [41]. Higher levels of 9CRA were observed in mice during the fasting state compared to the fed state. Additionally, in that study, they observed that tissue extraction methods significantly impact 9CRA detection, explaining why in previous studies 9CRA levels were often below the quantification limit [41]. As the consensus now indicates that 9CRA is definitely present in the liver, the role of RXRA becomes ever more important as its main effector. RXRA is downregulated during MASLDas the liver becomes more fibrotic (Figure S9) [42]. Combined with our data, this accentuates the potential of 9CRA as a therapeutic to reverse fibrosis and stimulate hepatocyte function.

A drawback of our study is that the liver organoids presented consisted of only two cell types involved in the development of chronic liver disease, namely, hepatocytes and HSCs. To develop a more advanced and representative model, other cell types, like immune cells and endothelial cells, should also be incorporated. It has been shown that RXRA is crucial for the development of macrophages [43]. The potential impact of 9CRA on liver macrophage and endothelial cell differentiation requires further investigation. For other commonly used in vitro liver models, 9CRA supplementation could have clear benefits, such as prevention of the dedifferentiation and loss of function occurring in primary hepatocytes when cultured in monolayers [44]. Similarly, primary HSCs activate when cultured as mono-layers in plastic culture dishes [45]. Because 9CRA reduced the activation levels of iHSCs and it has shown anti-fibrotic effects in activated rat HSCs in vivo [46], it could also potentially keep primary human HSCs more quiescent in monolayer cultures. Given RXR’s central role in nuclear receptor signalling, co-treatment with (ant)agonists of other nuclear receptors could have synergistic effects [47,48], and it is thus tempting to speculate on the benefits of combining 9CRA with other nuclear receptor agonists to prevent dedifferentiation of primary hepatocytes and promote primary HSC quiescence. Although 9CRA improved iPSC-derived liver organoid maturity and functionality, a certain degree of cross-linked collagen remained present in the organoids, suggesting that there is still room for further improvement. Future studies will investigate whether 9CRA is also beneficial for iPSC-derived liver-like cultures using methods established by others [8,49,50].

In summary, this study established the benefits of 9CRA for iPSC-derived liver cell and organoid differentiations, resulting in better liver fibrosis modelling. Combined with previous research, our findings suggest that 9CRA may also improve primary human liver cell cultures and other iPSC-derived liver differentiation processes.

## Figures and Tables

**Table 1:** Key resources table.

REAGENT or RESOURCE	SOURCE	IDENTIFIER
Antibodies		
HNF4α (1/200)	Santa Cruz Biotechnology (Dallas, TX, USA)	Cat# Sc-8987
CYP3A4 (1/200)	Cypex limited UK (Dundee, UK)	Cat# MA5-17064
αSMA-Cy3 (1/100)	Merck (Darmstadt, Germany)	Cat# C6198
VIM (1/100)	Dako (Glostrup, Denmark)	Cat# M0725
COL1 (1/100)	SouthernBiotech (Birmingham, AL, USA)	Cat# 1310-01
PDGFRβ (1/100)	Abcam (Cambridge, UK)	Cat# ab32570
Donkey anti-Rabbit IgG (H + L) Alexa Fluor 647 (1/200)	Invitrogen (Waltham, MA, USA)	Cat# A31573
Goat anti-Mouse IgG (H + L) Alexa Fluor 488 (1/200)	Invitrogen	Cat# A11029
Goat anti-Rabbit IgG (H + L) Alexa Fluor 488 (1/200)	Invitrogen	Cat# A11008
Donkey anti-Mouse IgG (H + L) Alexa Fluor 647 (1/200)	Thermo Fisher Scientific (Waltham, MA, USA)	Cat# A31571
Donkey anti-Goat IgG (H + L) Alexa Fluor 488 (1/200)	Thermo Fisher Scientific	Cat# A11055
Chemicals, peptides, and recombinant proteins		
Biolaminin	Biolamina (Sundbyberg, Sweden)	Cat# LN521-05
Essential 8 flex medium	Thermo Fisher Scientific	Cat# A2858501
DPBS with calcium and magnesium	Thermo Fisher Scientific	Cat# 14040117
StemPro Accutase Cell Dissociation Reagent	Thermo Fisher Scientific	Cat# A11105-01
RevitaCell Supplement 100X	Gibco (Waltham, MA, USA)	Cat# A2644501
mTeSR1 complete medium	Stemcell Technologies (Vancouver, BC, Canada)	Cat# 85850
BMP	PeproTech (Cranbury, NJ, USA)	Cat# 120-05
aFGF	PeproTech	Cat# 100-17A
Retinol	Merck Group	Cat# R7632
Palmitic acid	Sigma-Aldrich (St. Louis, MO, USA)	Cat# P5585
9-cis-retinoic acid	Sigma-Aldrich	Cat# R4643
Dimethyl sulfoxide	Merck Group	Cat# D2650
Trypsin-EDTA 0.05%	Invitrogen	Cat# 25300-054
Dulbecco’s modified medium	Capricorn (Ebsdorfergrund, Germany)	Cat# CA DMEM-HPA
HEPES Buffer solution	Capricorn	Cat# CA HEP-B
Penicillin-Streptomycin	Gibco	Cat# 15140122
Foetal Bovine Serum	TICO Europe (Amstelveen, Netherlands)	Cat# FBSEU500
Doxycycline hyclate	Merck Group	Cat# D9891
Activin A	PeproTech	Cat# AF-120-14E
Wnt3a	PeproTech	Cat# 315-20
HGF	PeproTech	Cat# 100-39
Essential amino acids	Thermo Fisher Scientific	Cat# 11130036
Non-essential amino acids	Thermo Fisher Scientific	Cat# 11140035
L-Serine	Thermo Fisher Scientific	Cat# A11179.22
Transforming growth factor beta	Peprotech	Cat# 100-21C
Acetaminophen	Merck Group	Cat# A7085
IGL-1 organ preservation solution	Institut Georges Lopez (Lissieu, France)	Cat# IGU.IGL-1 REV07
Pronase E	Merck	Cat# 1074330005
Collagenase P	Roche (Basel, Switzerland)	Cat# 11 213873001
Nycodenz gradient	Axis Shield Diagnostics (Dundee, UK)	Cat# 1002424
DNase I	Roche	Cat# 10104159001
Percoll gradient	GE Healthcare (Chicago, IL, USA)	Cat# GE17-0891-02
Williams E medium	Gibco	Cat# A12176-01
Glutamax supplement (200mM)	Thermo Fisher Scientific	Cat# 35050038
EGF	PeproTech	Cat# 315-09
Formaldehyde solution	Merck Group	Cat# 1004965000
Triton-X100	Merck Group	Cat# T8787
Bovine serum albumin	Sigma-Aldrich	Cat# A2153
Fluorescence Mounting Medium	Dako	Cat# S302380-2
DAPI dilactate	Merck Group	Cat# D9564
Xylene	VWR International (Radnor, PA, USA)	Cat# 28973.363
2-Propanol	Merck Group	Cat# 59300
Methanol	VWR International	Cat# 20903.368
Sirius Red	Sigma-Aldrich	Cat# 365548
Fast Green FCF	Merck Group	Cat# F7258
Picric acid solution	VWR International	Cat# 84512.260
DPX mounting medium for histology	Merck Group	Cat# 06522
Fixable viability stain 780	BD Biosciences (Franklin Lakes, NJ, USA)	Cat# 565388
Critical commercial assays		
CellTiter-Glo Luminescent Cell Viability assay	Promega (Madison, WI, USA)	Cat# G7571
ReliaPrep RNA Cell Miniprep System	Promega	Cat# Z6012
M-MLV Reverse Transcriptase, RNase H Minus and Point Mutant	Promega	Cat# M5301
GoTaq qPCR Master Mix with BRYT Green Dye	Promega	Cat# A6002
P450-Glo CYP3A4 activity assay	Promega	Cat# V9001
QuantSeq 3’ mRNA-Seq V2 Library Prep Kit FWD with UDI 12 nt set B1	Lexogen (Vienna, Austria)	Cat# 192.96
Deposited data		
All RNAseq SRA files are available at NCBI under Password for reviewers onolugasdncbnyn This includes Normalized_counts and Raw_counts files	GEO ID: GSE292959	
All other data files are deposited at Zenodo	10.5281/zenodo.15095698	
Experimental models: cell lines		
iPSCs	Sigma-Aldrich	IPSC0028-1VL
HepaRG	Biopredic (Saint-Grégoire, France)	Cat# HPR116
Oligonucleotides		
*DCN* forward (CCAATATCACCAGCATTCCTC)	Integrated DNA Technologies (IDT, Coralville, IA, USA)	NA
*DCN* reverse (CTGCTGATTTTGTTGCCATC)	IDT	NA
*LRAT* forward (GCTGGGCTTTACCCCCTA)	IDT	NA
*LRAT* reverse (CGAATAATTATCTTCACAGTCTCACAA)	IDT	NA
*COL3A1* forward (GGAGCTGGCTACTTCTCGC)	IDT	NA
*COL3A1* reverse (GGGAACATCCTCCTTCAACAG)	IDT	NA
*COL1A1* forward (CCGGCTCCTGCTCCTCTTAGCG)	IDT	NA
*COL1A1* reverse (CGTTCTGTACGCAGGTGATTGGTGG)	IDT	NA
*LOXL2* forward (GGAGAGGACATACAATACCAAAGTG)	IDT	NA
*LOXL2* reverse (CCATGGAGAATGGCCAGTAG)	IDT	NA
*ALB* forward (TGGCACAATGAAGTGGGTAA)	IDT	NA
*ALB* reverse (CTGAGCAAAGGCAATCAACA)	IDT	NA
*CYP3A4* forward (TTCCTCCCTGAAAGATTCAGC)	IDT	NA
*CY3A4* reverse (GTTGAAGAAGTCCTCCTAAGCT)	IDT	NA
*CYP1A2* forward (CTGGGCACTTCGACCCTTAC)	IDT	NA
*CYP1A2* reverse (TCTCATCGCTACTCTCAGGGA)	IDT	NA
*CYP2C9* forward (CAGTCCCTGCAGCTCTCTTT)	IDT	NA
*CYP2C9* reverse (TGCACAGTGAAACATAGGAAACTC)	IDT	NA
*HNF4α* forward (CACGGGCAAACACTACGGT)	IDT	NA
*HNF4α* reverse (TTGACCTTCGAGTGCTGATCC)	IDT	NA
*GAPDH* forward (AGCCACATCGCTCAGACAC)	IDT	NA
*GAPDH* reverse (GCCCAATACGACCAAATCC)	IDT	NA
*RPL19* forward (ATTGGTCTCATTGGGGTCTAAC)	IDT	NA
*RPL19* reverse (AGTATGCTCAGGCTTCAGAAGA)	IDT	NA
*RGS5* forward (TTGCATGTGCCAGAAAGCAG)	IDT	NA
*RGS5* reverse (TACCCCTTGGGTTTCGATGC)	IDT	NA
*HHIP* forward (CCGAGGCCATATTCCAGGTTT)	IDT	NA
*HHIP* reverse (TGGAAAGCACAACCCACCAT)	IDT	NA
Software and algorithms		
Graphpad	10.3.1	https://www.graphpad.com/
Flowjo	10	https://www.flowjo.com/
Qupath	0.4.4	https://qupath.github.io/
R	4.3.1	www.r-project.org
DESeq2	1.40.2	https://bioconductor.org/packages/release/bioc/html/DESeq2.html
clusterProfiler	4.8.3	https://bioconductor.org/packages/release/bioc/html/clusterProfiler.html
GSVA	1.48.3	https://bioconductor.org/packages/release/bioc/html/GSVA.html
Seurat	4.1.0	https://satijalab.org/seurat/
Python	3.7.7	https://www.python.org/
pySCENIC	0.11.0	https://pyscenic.readthedocs.io/en/latest/
Other		
96-well ultra-low attachment U-bottom plates	Greiner Bio-One (Kremsmünster, Austria)	Cat# 650970
ROTILABO round sieve, 75, 0.5 mm	Roth (Karlsruhe, Germany)	Cat# A624.1
Glomax	Promega	Cat# GM3000
Nanodrop 2000 Spectrophotometer	Thermo Fisher Scientific	Cat# G870
QuantStudio 3 Real-Time PCR System	Applied Biosystems (Foster City, CA, USA)	Cat# 272310385
EVOS	Thermo Fisher Scientific	Cat# AMF7000
40 µm cell strainer	Fisher Scientific (Geel, Belgium)	Cat# 11587522

## Data Availability

RNAseq data generated in this study are deposited at NCBI; GEO ID: GSE292959. Data have been deposited at Zenodo and are publicly available as of the date of publication (DOI 10.5281/zenodo.15095698). Any additional information required to reanalyse the data reported in this paper is available from the corresponding author upon request.

## Supplementary Materials

The following supporting information can be downloaded at https://www.mdpi.com/article/10.3390/cells14130983/s1, Table S1: Composition of first and second perfusion buffer; Figure S1: Differentiation of iPSCs towards iHSCs and iHepatocytes in 2D; Figure S2: iPSC-derived liver organoids are more fibrotic and display less hepatocyte maturity than HepaRG/pHSC organoids; Figure S3: Genes related to normal metabolism are lacking in iHepatocytes; Figure S4: RXRA TF activity is specific for quiescent HSCs compared to other mesenchymal cells [17,51]; Figure S5: Flow cytometry gating strategy to determine retinol uptake of iHSCs; Figure S6: Transcriptomic analysis of iHepatocyte differentiation with and without 9CRA treatment; Figure S7: Comparison of iHepatocytes treated with 9CRA from day 4 versus day 8 of differentiation; Figure S8: Continuation of 2D 9CRA treatment in 3D hampers spheroid formation and growth; Figure S9: RXRA is downregulated in fibrotic livers [42].

## Abbreviations

The following abbreviations are used in this manuscript:

HSC	Hepatic stellate cell
iPSC	Induced pluripotent stem cell
iHep	Induced pluripotent stem cell-derived hepatoblast
pHSC	Primary human hepatic stellate cell
iHSC	Induced pluripotent stem cell-derived hepatic stellate cell
iHepatocyte	Induced pluripotent stem cell-derived hepatocyte
9CRA	9-cis-retinoic acid
LDM	Liver differentiation medium
DMSO	Dimethyl sulfoxide
DOX	Doxycycline hyclate
TGFβ	Transforming growth factor beta
APAP	Acetaminophen
BSA	Bovine serum albumin
FBS	Foetal bovine serum
GSVA	Gene set variation analysis
RXRA	Retinoid X receptor alpha
