# Supplementary material for: 9-cis-Retinoic Acid Improves Disease Modelling in iPSC-Derived Liver Organoids
Source: Cells. 2025 Jun 26;14(13):983. doi: 10.3390/cells14130983 (PMC12249327; doi:10.3390/cells14130983)
Supplement: Supplementary file 1 [file cells-14-00983-s001.zip › cells-3686757-supplementary.pdf]

Supplemental information

Table S1. Composition of first and second perfusion buffer.

Perfusion buffer components (mg/L)	1st buffer	2nd buffer
NaCl	8000	8000
KCl	400	400
NaH_2PO_4<br>$NaH_2PO_4.12H_2O$	88<br>75.5	88<br>75.5
Na_2HPO_4	120.5	120.5
HEPES	2380	2380
$NaHCO_3$	350	350
EGTA	190	0
$CaCl_2.2H_2O$	0	560
D-(+)-Glucose	900	0
Phenol Red	6	6

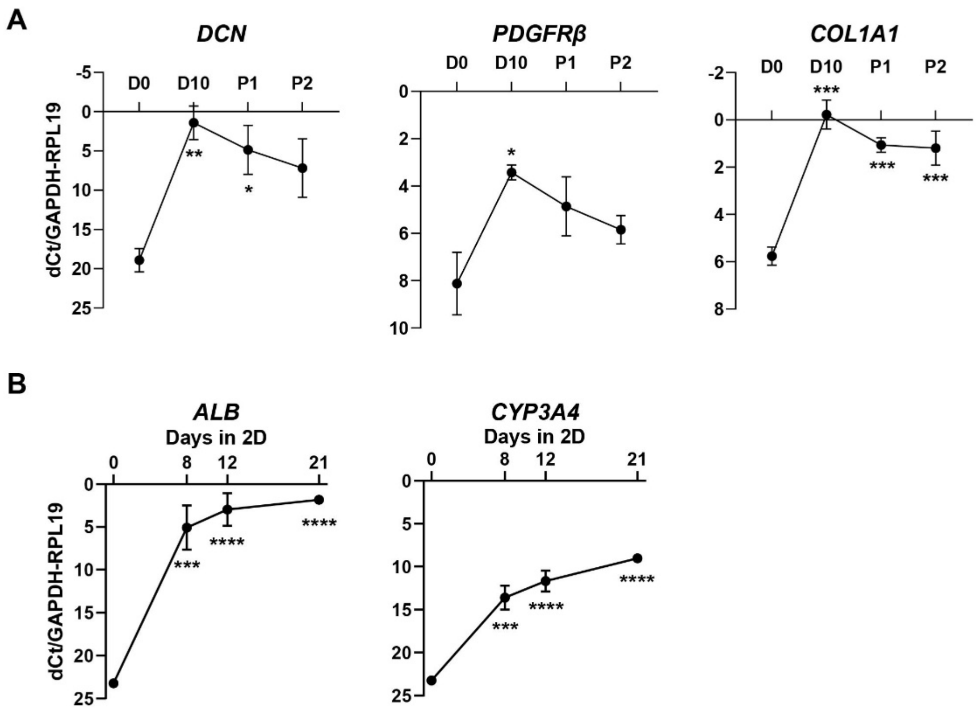

Figure S1. Differentiation of iPSCs towards iHSCs and iHepatocytes in 2D, related to Figure 1. A. Gene expression levels of the HSC markers DCN, PDGFRβ and COL1A1 during the iHSC differentiation. n = 3, * $p < 0.05$, ** $p < 0.01$, *** $p < 0.001$. **B.** Gene expression levels of the hepatocyte markers ALB and CYP3A4 during the iHepatocyte differentiation. n = 3, *** $p < 0.001$, **** $p < 0.0001$. All data are represented as mean ± SEM

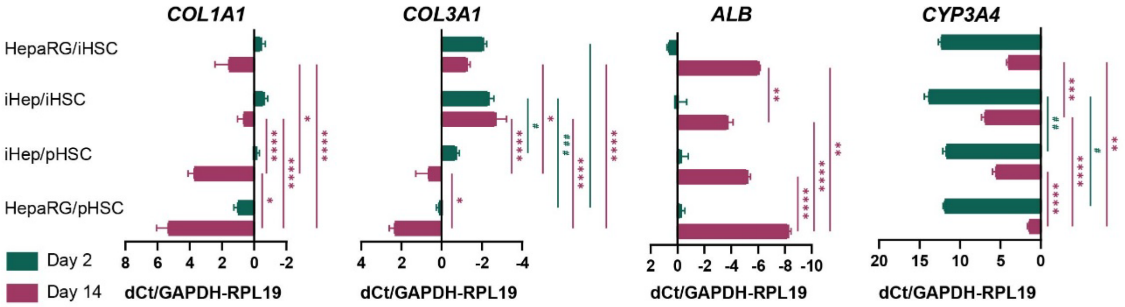

Figure S2. iPSC-derived liver organoids are more fibrotic and display less hepatocyte maturity than HepaRG/pHSC organoids, related to Figure 1. Gene expression levels of HSC activation genes *COL1A1* and *COL3A1,* and hepatocyte genes *ALB* and *CYP3A4* on day 2 and day 14 of culture. dCt values were calculated by subtracting the average Ct values of the reference genes from the Ct values of the gene of interest. n = 3-5, with 6 spheroids per repeat, * or # $p < 0.05$, ** or ## $p < 0.01$, *** or ### $p < 0.001$, **** $p < 0.0001$. Data are represented as mean ± SEM.

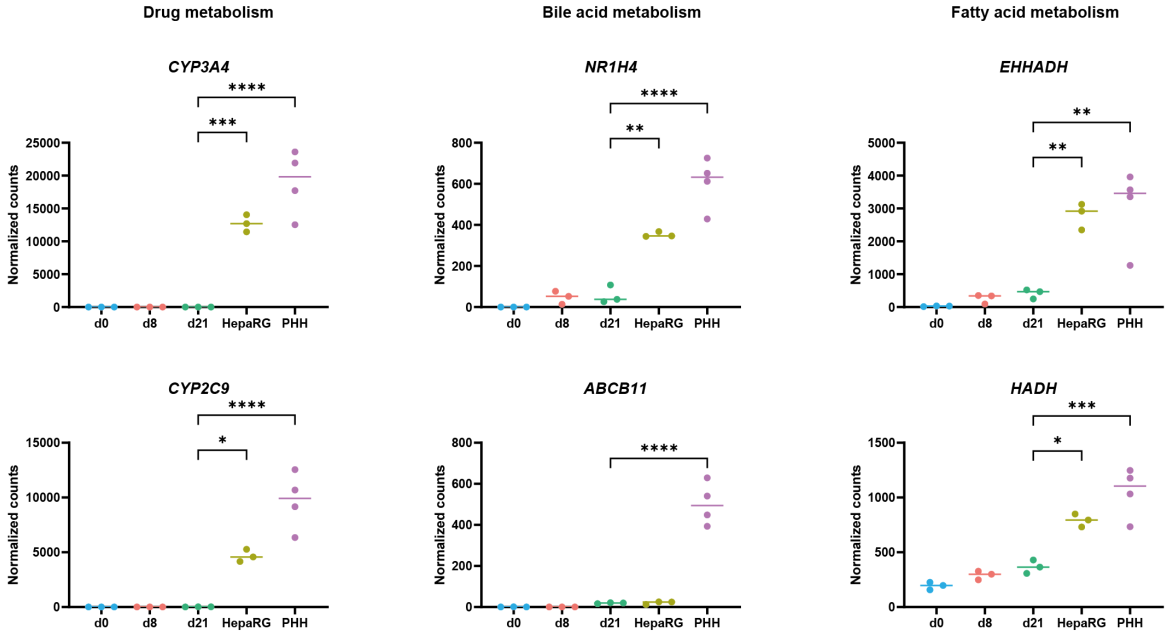

Figure S3. Genes related to normal metabolism are lacking in iHepatocytes, related to Figure 2. Gene expression levels of drug-, bile acid- and fatty acid metabolism markers during iHepatocyte differentiation, in HepaRG's and in primary hepatocytes. n = 3-5, * $p < 0.05$, ** $p < 0.01$, *** $p < 0.001$, **** $p < 0.0001$.

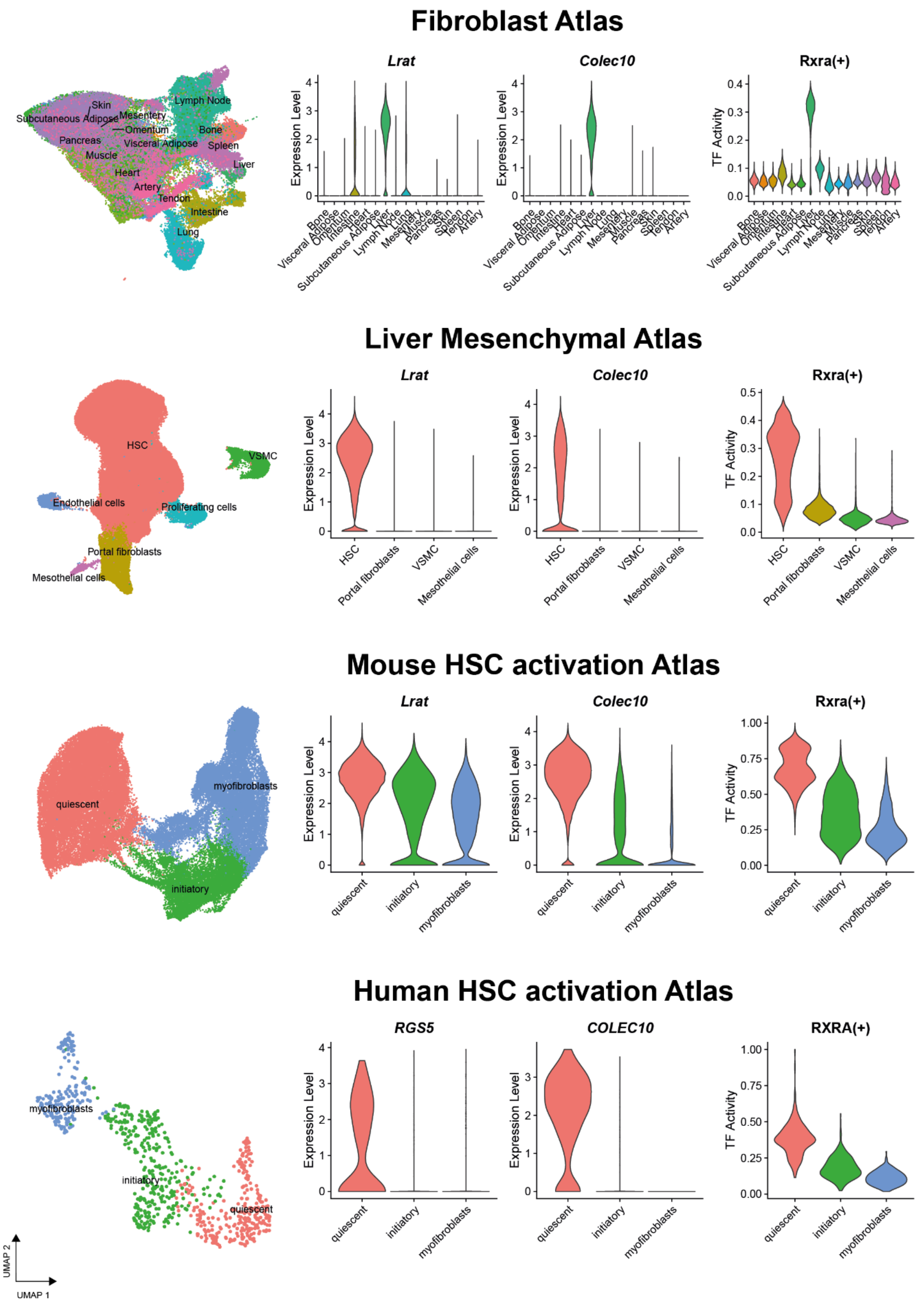

Figure S4. RXRA TF activity is specific for quiescent HSCs compared to other mesenchymal cells, related to Figure 3. Normalized expression of canonical quiescent HSC markers (*Lrat*, *Colec10*, *Rgs5*) and inferred RXRA activity in different mesenchyme focused atlases, including a mouse fibroblast atlas consisting of *Pdgfra* and *Dpt* positive cells from multiple organs, a mouse liver mesenchymal atlas consisting of *Pdgfrb* positive cells from the liver as well as the mouse and human HSC atlases[1,2].

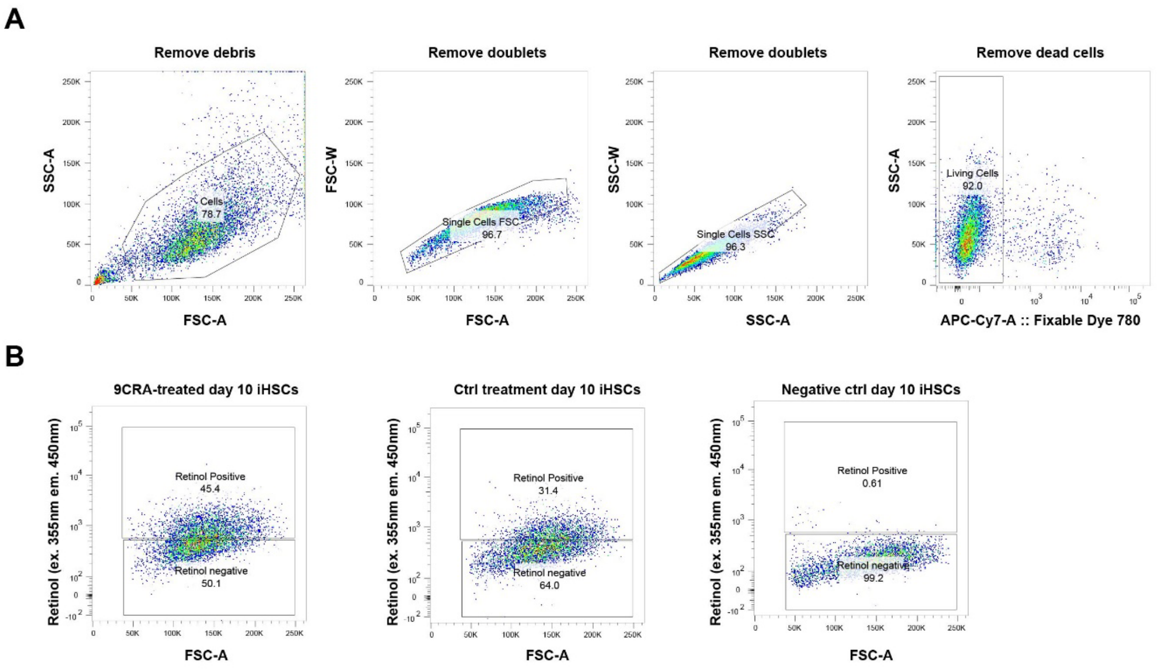

Figure S5. Flow cytometry gating strategy to determine retinol uptake of iHSCs, related to Figure 4. A. To eliminate debris, the forward scatter area (FSC-A) was plotted against the side scatter area (SSC-A). To remove doublets, first FSC-A then SSC-A were plotted against respectively forward scatter width (FSC-W) and side scatter width (SSC-W). Dead cells were excluded by removing those that stained positive for the fixable viability dye APC-Cy7. **B.** On day 10 of the differentiation 9CRA-treated versus control iHSCs were analysed for retinol uptake based on retinol autofluorescence (excitation wave length: 355 nm, emission wave length: 450 nm). As a negative control, iHSCs differentiated without retinol were used.

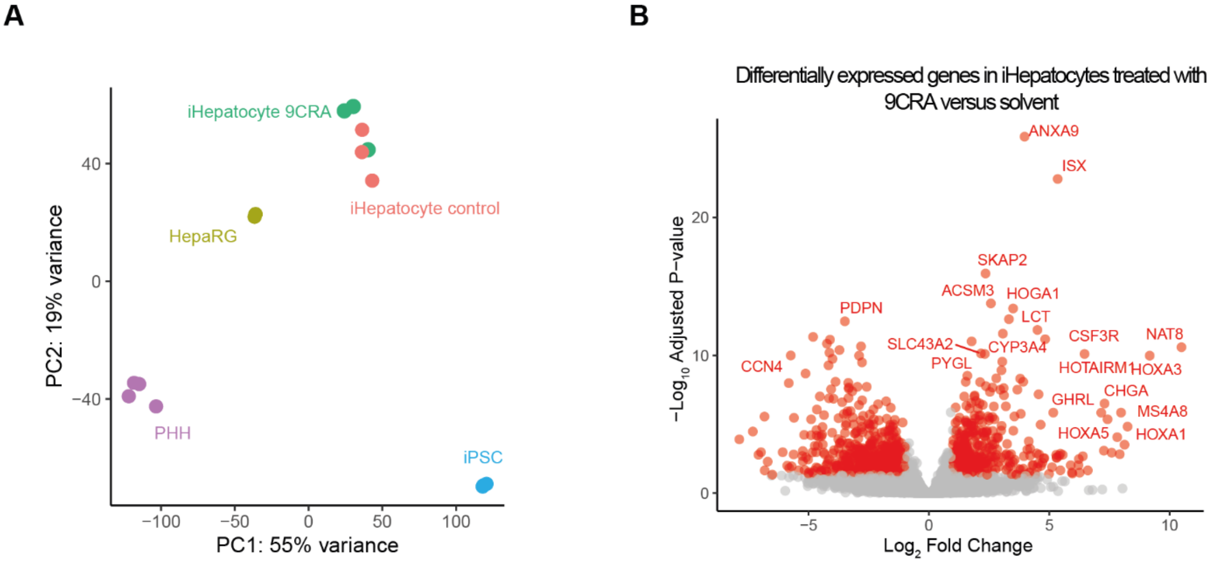

Figure S6. Transcriptomic analysis of iHepatocyte differentiation with and without 9CRA treatment , related to Figure 5. A. Principal component analysis of the transcriptomes of iHepatocytes after treatment with 9CRA or solvent as well as iPSCs, HepaRGs and freshly isolated primary human hepatocytes (PHH). **B.** Volcano plot of differentially expressed genes in 9CRA treated hepatocytes compared to solvent treated iHepatocytes (significant genes with foldchange > 2 or <-2 and adjusted p-value < 0.05 are indicated in red).

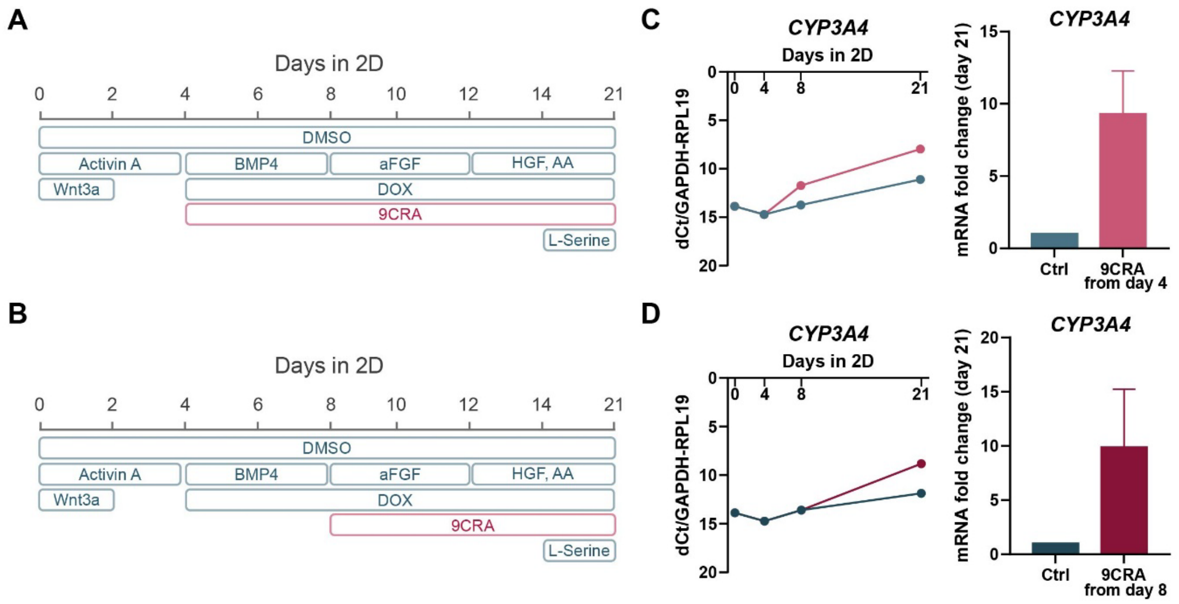

Figure S7. Comparison of iHepatocytes treated with 9CRA from day 4 versus day 8 of the differentiation, related to Figure 5. A. Schematic overview of the iHepatocyte differentiation protocol with the addition of 2 µM 9CRA from day four of the differentiation onwards. **B.** Gene expression levels of the mature hepatocyte gene *CYP3A4* over time with the addition of 9CRA from day four of the differentiation. Bar plots represent fold changes calculated using the untreated cells as a control (n=2). **C.** Schematic overview of the iHepatocyte differentiation protocol with the addition of 2 µM 9CRA from day eight of the differentiation onwards. **D.** Gene expression levels of the mature hepatocyte gene *CYP3A4* over time with the addition of 9CRA from day eight of the differentiation. Bar plots represent fold changes calculated using the untreated cells as a control (n=2). Data are represented as mean ± SEM.

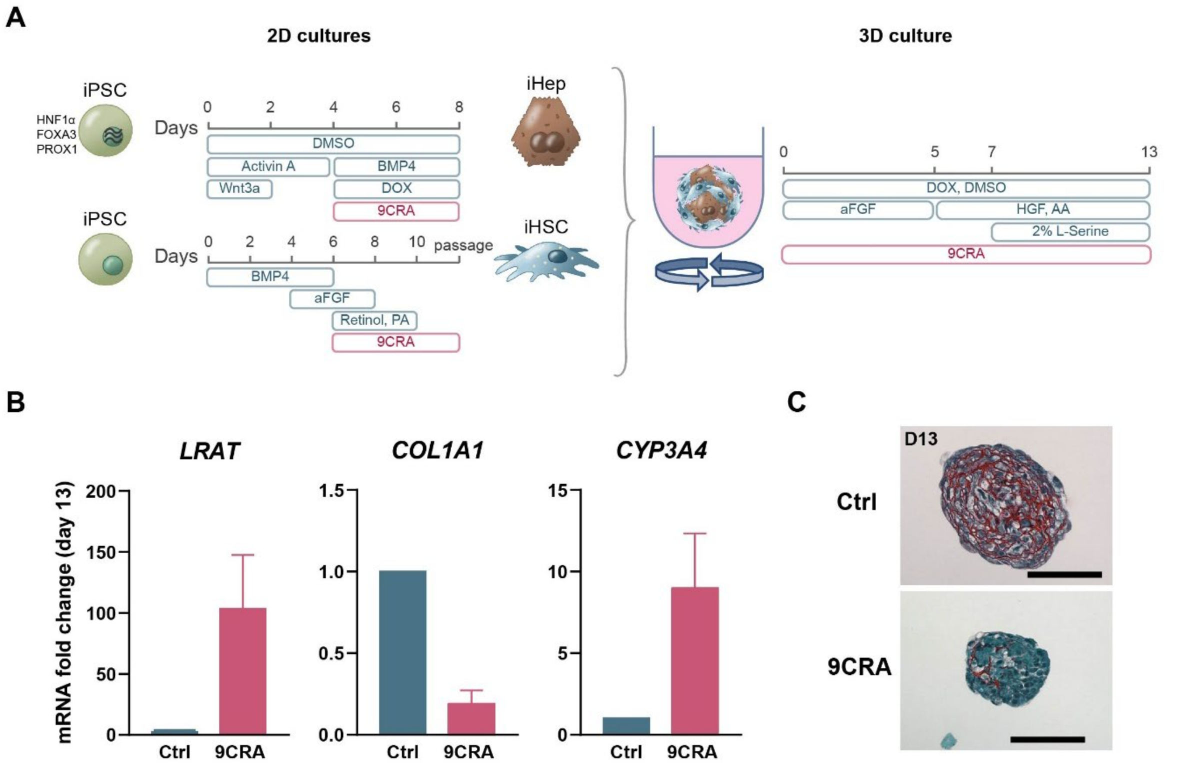

Figure S8. Continuation of 2D 9CRA treatment in 3D hampers spheroid formation and growth, related to Figure 6. A. Schematic representation of the differentiation of iPSCs towards iHeps and iHSCs with the addition of 2 µM 9CRA, and their incorporation into spheroid cultures with continuation of 2 µM 9CRA during the entire 3D culture. **B.** Gene expression levels of the quiescent HSC gene *LRAT*, the HSC activation gene *COL1A1* and the hepatocyte gene *CYP3A4*, at the end of the spheroid culture with or without the addition of 9CRA. n= 2, with 6 spheroids per repeat. Data are represented as mean ± SEM. **C.** PSR staining on day 13 of the spheroid culture. Scale bars represent 100 µm.

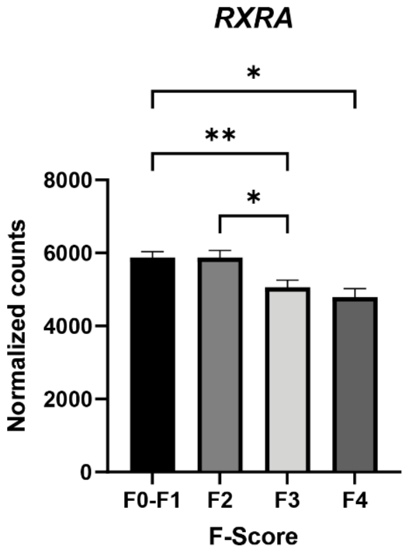

Figure S9. RXRA is downregulated in fibrotic livers, related to discussion. Normalized expression of RXRA in patients with different stages of liver fibrosis[3], n = 216.

References

1. Merens, V., Knetemann, E., Gurbuz, E., De Smet, V., Messaoudi, N., Reynaert, H., Verhulst, S., and van Grunsven, L.A. (2025). Hepatic stellate cell single cell atlas reveals a highly similar activation process across liver disease aetiologies. JHEP Rep *7*, 101223. 10.1016/j.jhepr.2024.101223.
2. Buechler, M.B., Pradhan, R.N., Krishnamurty, A.T., Cox, C., Calviello, A.K., Wang, A.W., Yang, Y.A., Tam, L., Caothien, R., Roose-Girma, M., et al. (2021). Cross-tissue organization of the fibroblast lineage. Nature *593*, 575-579. 10.1038/s41586-021-03549-5.
3. Govaere, O., Cockell, S., Tiniakos, D., Queen, R., Younes, R., Vacca, M., Alexander, L., Ravaioli, F., Palmer, J., Petta, S., et al. (2020). Transcriptomic profiling across the nonalcoholic fatty liver disease spectrum reveals gene signatures for steatohepatitis and fibrosis. Sci Transl Med *12*. 10.1126/scitranslmed.aba4448.
